# Biotechnological and Agronomic Potential of Endophytic Pink-Pigmented Methylotrophic *Methylobacterium* spp.

**DOI:** 10.1155/2015/909016

**Published:** 2015-03-10

**Authors:** Manuella Nóbrega Dourado, Aline Aparecida Camargo Neves, Daiene Souza Santos, Welington Luiz Araújo

**Affiliations:** Department of Microbiology, Institute of Biomedical Sciences, University of São Paulo, Brazil

## Abstract

The genus *Methylobacterium* is composed of pink-pigmented facultative methylotrophic (PPFM) bacteria, which are able to synthesize carotenoids and grow on reduced organic compounds containing one carbon (C_1_), such as methanol and methylamine. Due to their high phenotypic plasticity, these bacteria are able to colonize different habitats, such as soil, water, and sediment, and different host plants as both endophytes and epiphytes. In plant colonization, the frequency and distribution may be influenced by plant genotype or by interactions with other associated microorganisms, which may result in increasing plant fitness. In this review, different aspects of interactions with the host plant are discussed, including their capacity to fix nitrogen, nodule the host plant, produce cytokinins, auxin and enzymes involved in the induction of systemic resistance, such as pectinase and cellulase, and therefore plant growth promotion. In addition, bacteria belonging to this group can be used to reduce environmental contamination because they are able to degrade toxic compounds, tolerate high heavy metal concentrations, and increase plant tolerance to these compounds. Moreover, genome sequencing and omics approaches have revealed genes related to plant-bacteria interactions that may be important for developing strains able to promote plant growth and protection against phytopathogens.

## 1. *Methylobacterium* Genus

**Figure 1:**
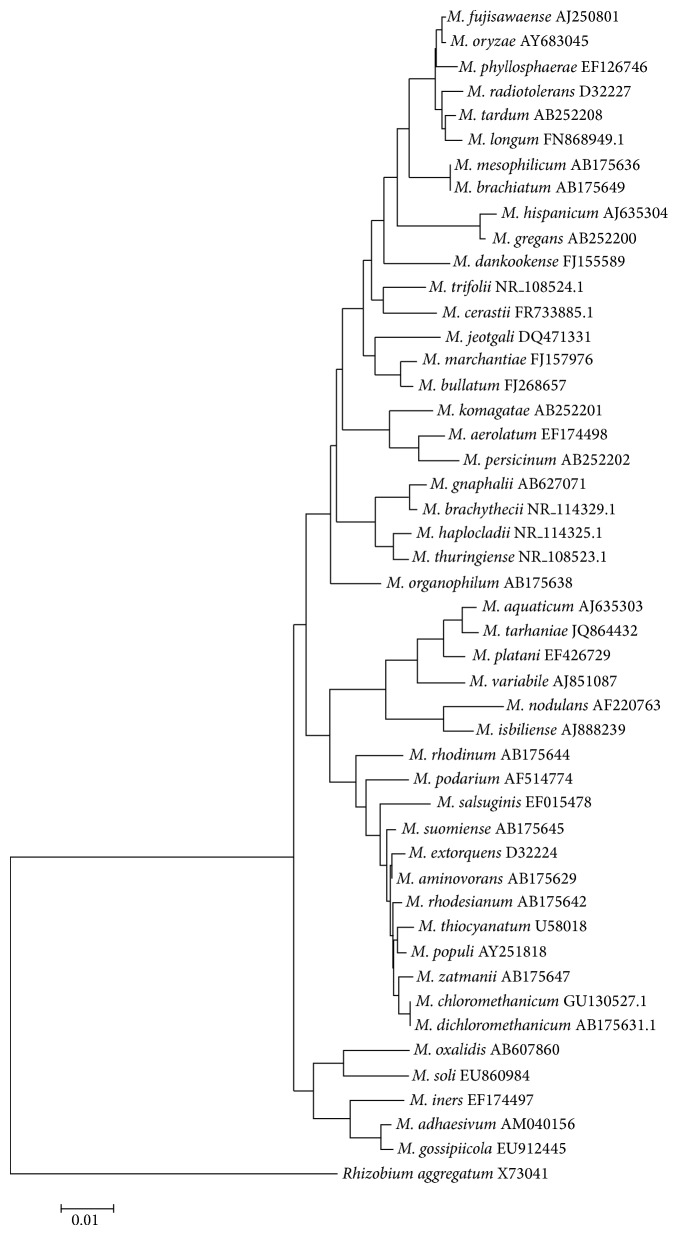
Phylogenetic analysis of the 16S* rDNA* genes of* Methylobacterium* spp. strains (sequences available in Ribosomal Database Project query and the NCBI database) using the Kimura model. There were a total of 1288 nucleotide positions in the final dataset, and* Rhizobium aggregatum* served as an outgroup.

Members of the* Methylobacterium* genus are classified as *α*-proteobacteria and include 51 reported species (http://www.bacterio.net/methylobacterium.html), which are represented in Figure 1; most of them (35) were reported in the last 10 years and are closely phylogenetically related (Figure 1).* M. organophilum* is the type strain [1], although* M. extorquens* strain AM1 (Table 1), isolated as an airborne contaminant growing on methylamine [2], is the most studied strain and has been used as a model organism for this genus, including for plant interactions and methylotrophic metabolism studies.

This genus is composed of Gram-negative bacteria that are generally with pink pigmentation due to carotenoid synthesis [3], rod shaped, strictly aerobic and able to grow using compounds containing only one carbon (C_1_), such as methanol and methylamine [4]. Thus, these bacteria are denoted “pink-pigmented facultative methylotrophs” (PPFMs). The main characteristic of this group is the ability to oxidize methanol using the methanol dehydrogenase enzyme (MDH). The* mxa*F gene encodes the large subunit of this enzyme, which is key in methylotrophic metabolism and is used to study that group of bacteria [5, 6].

Members of the* Methylobacterium* genus occupy different habitats due to their great phenotypic plasticity, including soil, water, leaf surfaces, nodules, grains, and air [7–9]. They can also be opportunistic pathogens in human beings [10]. Growing in meristematic tissue, they can reach populations of 10^4^ to 10^6^ colony-forming units (CFU) per gram of plant tissue [11]. In addition, they can form biofilms [12, 13] and use methylotrophic metabolism as an adaptive advantage during plant host colonization [14].

The association between* Methylobacterium* spp. and host plants varies from strong or symbiotic [15] to weak or epiphytic [16] and to intermediate or endophytic [17]. During interactions with plants,* M. nodulans* and* M. radiotolerans* have been reported to be involved in nitrogen fixation and nodule formation [18, 19], while other* Methylobacterium* species may be related to phytohormone production [20] or interacting with plant pathogens [17, 21], promoting plant growth [22, 23] and inducing higher photosynthetic activity [24].

Although* Methylobacterium* spp. are not phytopathogenic bacteria and are not associated with the degradation of plant biomass (saprophytes), it was reported that some strains are able to synthesize pectinase and cellulase, suggesting they can induce systemic resistance during plant colonization [22, 25]. In addition, they can help plant development by decreasing environmental stress and by degrading toxic organic compounds [26], immobilizing heavy metals [27], or even inhibiting plant pathogens [28]. Thus, these bacteria can play an important role in the microbial balance in plants, highlighting their importance during plant development.

Therefore, this review aims to give an overview of reported genes and proteins involved in* Methylobacterium*-plant interaction process, not only the mechanisms that induce plant growth, systemic resistance, and plant pathogen inhibition but also involved in the bioremediation of organic (herbicides) and inorganic (heavy metals) compounds from contaminated crop soil or water that can be used to irrigate the agriculture soil. The present review also comprises the studies that use genomic, transcriptomic, proteomic, and metabolomics to identify the genes and proteins potentially involved in this plant interaction. The comprehension of how this* Methylobacterium*-plant interaction occurs can allow us to increase crop production and decrease environmental pollution, possibly creating a biotechnological product. Therefore, besides an agronomic application, other biotechnological uses of this genus will also be reported during this review showing the potential of these facultative pink-pigmented methylotrophic genera.

## 2. *Methylobacterium* during Plant Interactions

Bacteria may interact with plants, acting as pathogens, epiphytes or endophytes. Endophytic microorganisms are defined as those which live part of or whole life inside the plant tissues without causing damage or forming visible external structures to the host [29, 30]. Thus, this definition excludes mycorrhizal fungi, nodulating symbiotic bacteria, epiphytic microorganisms, and phytopathogens [31]. Depending on environmental conditions, a microorganism classified as an endophyte can behave like an epiphyte or a subclinical pathogen [32]; in some cases, the presence of an endophyte can be associated with the presence of a pathogen or another microorganism, between which there may be an intimate interaction [21].

*Methylobacterium* spp. can be found in association with more than 70 species of plants [16], actively colonizing the root, branches and leaves [12] with several studies reporting* Methylobacterium* as a putative endophyte of different host plants, such as cotton [7], peanut [22], citrus [21], pinus [33], eucalyptus [34], sunn hemp [18],* Catharanthus roseus*, tobacco [12], strawberry [35], and mangrove plants [27]. Knief et al. [36] reported based on the metagenomics and metaproteomics approach that alphaproteobacterium are found more frequently in rice phylosphere than in rice rizosphere, where the most abundant genera are* Rhizobium* and* Methylobacterium* indicating that these genera prefer the host plant environment. Furthermore, within this genus there is a diversity of* Methylobacterium* species inside the plant host [21, 34].

The first step of the plant-bacteria interaction is the recognition of plant exudates by the bacteria. Such exudates are composed mainly of sugars, amino acids, and organic acids as well as flavonoids [37, 38], and they are able to attract specific and beneficial microorganisms [39], establishing an indwelling bacteria-plant interaction. Root exudates possibly influence host recognition, biofilm formation, and colonization by* Methylobacterium* spp. as endophytes [13]. After plant recognition, surface colonization likely depends on traits such as adhesins, pili, and EPS (exopolysaccharides) to attach to the cells on the surface. Kwak et al. [40] reported the presence of ten genes involved in type-IV pilus biosynthesis and two genes related to hemolysin-type adhesins in* M. oryzae* CBMB20. Numerous studies have reported* Methylobacterium* spp. cells colonizing plant tissues in a mucilaginous layer [12, 13, 41], suggesting that this could be a first step during plant colonization.

Thus, the endophytic bacteria are able to penetrate into the plant, generally through root wounds, and systemically colonize the host, inhabiting the apoplast [42], conductive vessels [42, 43] and occasionally the intercellular space [44]. However, colonization is somehow guided toward plant-bacteria-specific interactions. Poonguzhali et al. [41] observed intercellular colonization in tomato roots by* M. suomiense* CBMB120, but not in rice. Through systemic colonization, these bacteria can change the physiological and morphological conditions of the host, and they can affect the populations of other microorganisms present in the plant [12]. Reinhold-Hurek and Hurek [30] reported that endophytic bacteria may influence the physiology of the host plant by processes that are not yet clarified. This influence is due to the close relationship between the different individuals who developed during the coevolution of species. Bacteria present several properties of interest within their hosts, protecting them from insect attacks, pathogens, and herbivores, producing plant growth hormones, and providing more nutrients and biological nitrogen fixation [45].

Plant colonization by the endophytes may be guided by some quorum sense (QS) systems, using signaling molecules commonly found in Gram-negative bacteria, such as N-acyl-homoserine lactones (AHLs), which are regulated by the LuxI/LuxR system [46]. Bacteria can work as a multicellular organism due to the QS system once the bacterial population growth and the extracellular concentration of AHLs increases, reaching a level that can regulate the transcription of different genes that may be related to the secretion system, biofilm formation, exchanges of DNA and others [47].* Methylobacterium* strains produce AHL molecules [48], which are responsible for bacterial cell-to-cell communication [43] and are produced with an increase in bacterial cell density. This molecule, in* Methylobacterium* sp. strain GXF4, may influence bacterial communication and colonization outcomes within the xylem [43], indicating that* Methylobacterium* strains may also interact with other microorganisms inside the plant, including phytopathogens such as* Xylella fastidiosa* [49].* M. mesophilicum* SR1.6/6, isolated from the interior of the sweet orange tree (*Citrus sinensis*) [21], is also able to produce six long-chain homoserine lactones (HSLs) (the saturated homologs (*S*)-*N*-dodecanoyl and (*S*)-*N*-tetradecanoyl-HSL, the uncommon odd-chain* N*-tridecanoyl-HSL, the new natural product (*S*)-*N*-(2*E*)-dodecenoyl-HSL, and the rare unsaturated homologs (*S*)-*N*-(7*Z*)-tetradecenoyl, and (*S*)-*N*-(2*E*,7*Z*)-tetradecadienyl-HSL), as described by Pomini et al. [48]. For this strain, the* (S)-N-*dodecanoyl-HSL molecule was able to upregulate the expression of genes related to plant-bacteria interactions, such as bacterial metabolism (*mxa*F), adaptation to stressful environments (*crt*I and* sss*), interactions with plant metabolism compounds (*acd*S) and pathogenicity (*patatin*), showing that the AHL molecule, together with bacterial density, activates several plant-bacteria interaction genes [50]. These results imply that, although* Methylobacterium* spp. are able to identify the plant exudate and trigger a response, during plant colonization, the gene coordination may also be regulated by a quorum-sensing system to allow efficient plant colonization.

### 2.1. The Role of Methylotrophic Metabolism during Plant Interactions

Methylotrophic bacteria, including* Methylobacterium* spp., were first reported by Anthony [51]. These bacteria are able to use (C1) compounds (mainly methanol but also formaldehyde and formate [7]) as a sole carbon source, or they can use multicarbon compounds with or without carbon-carbon bonds.* Methylobacterium* spp. can also use other carbon sources: those with two carbons, such as acetate, ethanol, and ethylamine; those with three carbons, such as pyruvate; or those with four carbons, such as succinate [52]. This ability to use several carbon sources allows* Methylobacterium* spp. to colonize different environments, including plants, which release methanol by stomata during plant growth [35].

**Figure 2:**
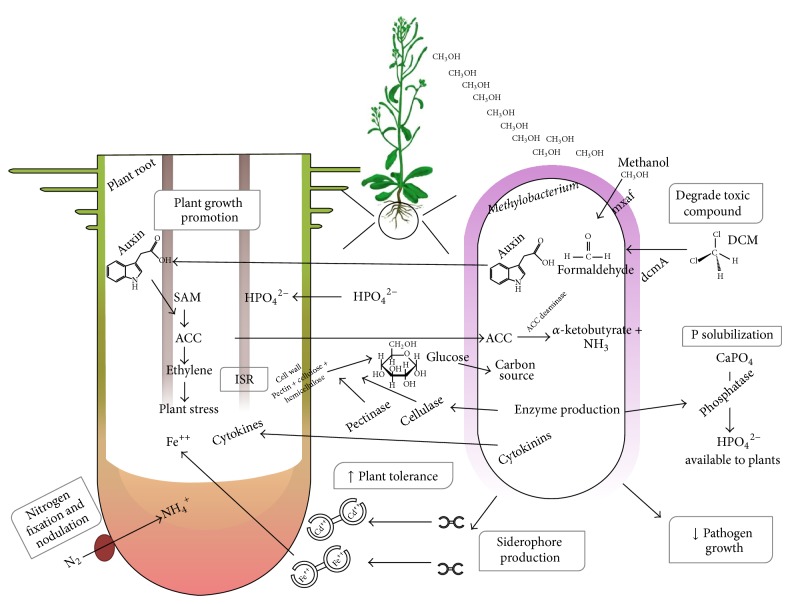
Molecular mechanisms possibly involved in plant colonization and plant growth promotion identified in* Methylobacterium* spp. genomes. The molecular communication during plant-*Methylobacterium* species interaction involves bacterial proteins and the secretion of phytohormones (auxin and cytokinin) that induce plant growth and decrease pathogen growth.* Methylobacterium* spp. are able to modulate ethylene levels using ACC (an ethylene precursor) as a source of nitrogen. The auxin and ethylene pathways are related, whereas ACC plant production is induced by bacterial auxin.* Methylobacterium* spp. can also solubilize phosphorus and produce siderophores that can chelate iron and other metals recognized and absorbed by the plant, increasing nutrient uptake (mainly Fe). The bioremediation of toxic organic compounds is also be observed during DCM degradation, as is the chelation of inorganic toxic compounds, increasing plant tolerance. Methylotrophic metabolism bacteria present an adaptive advantage due to methanol exudation by plant leaves. These molecular processes modulate* Methylobacterium* spp. plant colonization and plant defense.

Bacterial methylotrophic metabolism starts in the periplasm, where the methanol dehydrogenase (MDH) enzyme oxidizes methanol into formaldehyde (Figure 2). MDH is an *α*_2_ *β*_2_ tetramer with two active sites, a prosthetic group and a calcium atom [5]. The large and small subunits are encoded by the* mxa*F and* mxa*I genes, respectively; moreover,* mxa*G encodes cytochrome c, the primary electron acceptor for MDH [53]. MDH is composed of two large (66 kDa) and two small (8.5 kDa) subunits. The large subunit (MxaF) is essential for methanol dehydrogenase activity; it contains a PQQ prosthetic group [54]. This enzyme is a soluble quinoprotein that uses pyrroloquinoline quinone (PQQ) as a cofactor to transfer two electrons to cytochrome c [53]. Methanol oxidation generates formaldehyde (a main intermediate of methylotrophic metabolism), which can also be assimilated in the serine cycle and used by the cell or oxidated to CO_2_, generating energy; each molecule of methanol generates 1 ATP [54].

Chistoserdova et al. [55] observed that* M. extorquens* strain AM1 contains 70 genes that are related to methylotrophic metabolism. These genes are distributed into eight regions of the bacterial chromosome. One 12.5 kb cluster contains 14 genes transcribed in the same direction (*mxa*FJGIRSACKLDEHB), and upstream of this cluster there is the* mxa*W gene transcribed in the opposite direction. There are also the transcriptional regulators* mxb*MD and* mxc*QE [55]. Moreover, the* M. extorquens* AM1 genome contains two copies of the* xox*F gene, which encodes a PQQ-dependent periplasmic alcohol dehydrogenase that is 50% identical to* mxa*F. When both* xox*F genes are absent, the strain lacks methanol dehydrogenase activity and losses both the ability to grow in methanol as the sole carbon source and the* mxa* promoter, suggesting that* xox*F is responsible for the regulatory complex [54] that influences methanol metabolism.

The major source of methanol in the atmosphere is forests, due to plant emissions [56] because during plant growth, cell expansion depends on pectin breakdown by pectin methylesterase, resulting in the production of methanol that is released by stomata [35]. Methanol production may fluctuate according to environmental conditions, such as flooding [57], plant age [52], and physiological state because in mature (yellow) leaves and during abscission, methanol release increases significantly [58]. In this way, during plant colonization,* Methylobacterium* spp. may take advantage of the presence of methanol released by plants by expressing genes related to methylotrophy [59], such as* mxa*F, colonizing the host plant more efficiently than other plant-associated bacteria. In fact,* mxa*F*-*defective* M. extorquens* mutants were less competitive than the wild-type strain during the colonization of* Medicago truncatula* under competitive conditions [14]. The authors observed that the ability to use methanol as a carbon and energy source provided a selective advantage during host colonization. However, after a single colonization the defective mutants were able to colonize the plant tissues, suggesting that methanol is not the only carbon source that is used by* Methylobacterium* spp. during endophytic and epiphytic plant colonization.

### 2.2. Induction of Plant Growth

The cascade of events that occurs after a bacterial cell recognizes plant exudates results in major changes in cellular metabolism, including the accumulation of several secondary metabolites [45]. Such physiological changes can modulate the growth and development of the plant. However, considering their complexity, these mechanisms and networks are still far from being elucidated. Therefore, different studies have been conducted with the aim of uncovering these mechanisms.

Plant growth stimulation by endophytic bacteria is largely due to phytohormone production, and several studies have been reported the interaction of* Methylobacterium* species with different plant species by regulating phytohormone production [60].* Methylobacterium* strains have been reported to produce phytohormones such as cytokinins and auxins [61], which promote cell division and elongation, respectively. Pirttilä et al. [62] tested for the most common gibberellin production in* M. extorquens*, but such compounds were not found. Instead, the bacterium produced adenine derivatives that may be used as precursors in cytokinin biosynthesis. In a recent study, Kwak et al. [40] showed that the* M. oryzae* CBMB20 features two* mia*A genes, which are critical for the production of zeatin, a major cytokinin. Another important hormone is auxin. The main auxin in plants is indole-3-acetic acid (IAA), which controls an important physiological process during root development [63]. In the* Methylobacterium* genus, genes that encode enzymes related to auxin biosynthesis, such as amine oxidase, aldehyde dehydrogenase, nitrilase/cyanide hydratase, N-acyltransferase, nitrile hydratase, amidase, have been reported [22, 23, 40].* Methylobacterium* is able to produce IAA [64], suggesting that its inoculation can increase plant IAA concentrations and induce plant growth promotion [65].

Another compound that regulates root growth and development is ethylene [66], which is related to the auxin biosynthesis pathway [39]. High concentrations of ethylene are related to stress conditions in plants and may have a deleterious effect on plant growth, inhibiting root elongation and accelerating abscission, aging, and senescence [67]. In ethylene biosynthesis, the precursor of the ethylene hormone is ACC (aminocyclopropane-1-carboxylic acid), which is converted from S-adenosylmethionine (SAM) to ethylene by the actions of ACC synthase (ACS) and ACC oxidase (ACO), enzymes that are transcriptionally regulated independently by both biotic and abiotic factors [39, 66, 67]. Plant ACS activity can also be increased by bacterial IAA production, showing that both pathways are related, and may increase plant and bacterial fitness during* Methylobacterium*-plant interactions (Figure 2). There are also suggestions that the amount of IAA released may have an important role in modulating plant-microbe interactions, and the balance between IAA and ethylene might be fundamental for the maintenance of endophytic bacterial plant colonization, as proposed by Hardoim et al. [39].

Moreover, endophytes carry several important genes related to beneficial interactions with the host plants, including the* acd*S gene [39]. The* acd*S gene encodes an ACC deaminase enzyme that converts ACC into ammonia (NH_3_) and *α*-ketobutyrate. An analysis of the genomes of* Methylobacterium* species revealed that the plant-associated species, such as* M. oryzae*,* M. nodulans*, and* M. radiotolerans*, contain this ACC deaminase gene [40] and that* M. nodulans* and* M. radiotolerans* are able to use ACC as a nitrogen source by the actions of ACC deaminase, reducing ethylene levels [60] and consequently the stress ethylene response in the host plant. Joe et al. [68] reported that the association between an ACC deaminase-positive* M. oryzae* CBMB20 strain with* Azospirillum brasilense* CW903 strain reduced ethylene levels in plants. These authors developed coaggregated cell inoculants containing both strains, which improved plant resistance and reduced stress in inoculated tomato plants. Similar results were observed in canola: when a plant-growth promoting* Methylobacterium* containing ACC deaminase was inoculated into canola roots, it also reduced the concentrations of ACC and ethylene in the plant, increasing root length [65, 66]. Therefore, bacteria that are able to reduce ethylene levels in the host plant are associated with plant growth promotion [39, 60, 66].

In addition to phytohormone production,* Methylobacterium* presents other beneficial plant interactions, improving the cycling of nutrients such as siderophore production, which is important to increase the iron supply to the plant and to reduce heavy metal toxicity [69]; nitrogen fixation, which favors plant biomass increase [18, 70] and phosphate solubilization, making phosphate available for plant uptake [71]. All of these processes are considered to be involved in plant nutrient acquisition and are responsible for promoting plant growth.

Nitrogen is often the most limiting nutrient for plant growth, but nitrogen from the atmosphere is unavailable to plant metabolism. Thus, the process of nitrogen fixation involves the transformation of atmospheric nitrogen into ammonia, which is available for plant use. The biological reduction of nitrogen to ammonia can be performed only by some prokaryotes with the presence of the nitrogenase enzyme [19].* M. nodulans* was originally isolated from* Crotalaria podocarpa* [18] and together with* Methylobacterium* sp. 4-46 represents the few nodulating* Methylobacterium* species reported so far [40].* M. nodulans* ORS2060 was reported to contain the* nif*H gene (involved in nitrogen fixation) and to induce nitrogen-fixing nodules on the roots of legumes by the* nod*A gene [15]. Nodules are specialized organs in which fixing nitrogen bacteria reduce atmospheric nitrogen to ammonia [18].* M. nodulans* seems to have a competitive advantage during plant colonization and nodule formation because of its ability to obtain carbon both from sugars (host plant photosynthesis) and methanol (from methylotrophy) [72]. It was previously reported that the loss of the bacterial methylotrophic function significantly affected plant development because the inoculation of* M. nodulans* nonmethylotrophic mutants in* C. podocarpa* decreased the total root nodule number per plant and the whole-plant nitrogen fixation capacity, also reducing the total dry plant biomass compared with the wild-type strain [70].

Another essential nutrient present in the soil is phosphorus. Despite the high concentrations of total phosphor in soils, most of it is in the form of inorganic compounds bound to calcium, iron, or aluminum or immobilized in organic matter such as phytate (phytic acid, myo-inositol hexakisphosphate), the most abundant organic phosphorus compound in soil [73]; therefore, it is not available for plant uptake [74]. Single-cell organisms assimilate mainly soluble ionic phosphate forms (HPO_4_^2−^, H_2_PO_4_^−^), but the concentration of soluble phosphorus in soil is usually very low. There are considerable populations of phosphate-solubilizing bacteria in soil and in plant rhizospheres, which are important for increasing plant biomass by converting both organic and inorganic insoluble phosphate to a form available to plants [75]. Among those,* Methylobacterium* spp. have the ability to dissolve inorganic phosphates, which may be further involved in phosphate metabolism in both microorganisms and plants [76]. There are three different types of microbial enzymes that solubilize organic phosphate: nonspecific acid phosphatase, phytase and C-P lyase (or phosphonatase). They all release phosphate, the first from phosphoric ester or phosphoric anhydride, the second from phytic acid, and the third from organophosphonates.* M. oryzae* has been reported to have genes encoding all three phosphatase enzymes [40].

Another positive plant-bacteria interaction attribute is the ability to synthesize bacterial siderophores. Siderophores are low-molecular-mass compounds with a high affinity for iron that are produced by bacteria to solubilize iron to promote its efficient uptake. Iron is extremely necessary to almost all forms of life because it participates in numerous biological processes; however, it exists mainly as insoluble Fe^3+^ in aerobic environments [77]. Therefore, siderophore release is an effective strategy developed by bacteria for iron acquisition that can, in turn, make this metal also available for plant uptake, contributing to plant growth [78]. In the* Methylobacterium* genus, the iron uptake genes* iuc*A and* iuc*C have been described in 35 strains, including* M. extorquens* strains AM1, PA1, DM4, and CM4 and* M. populi* [23].

In a study using WC-MS (whole-cell matrix-assisted laser desorption ionization-time of flight mass spectrometry), siderophore production and phosphate solubilization were analyzed in 190 unique strains of* Methylobacterium* species collected from leaf samples of many host plants [79]. Among these* Methylobacterium* isolates, 185 (growing on methanol as a carbon source) and 93 (growing on glucose as a carbon source) strains were positive for calcium phosphate solubilization. Siderophore production was positive in 35 strains [79]. Lacava et al. [80] also evaluated siderophore production by 37* Methylobacterium* spp. strains and observed that all the tested strains were able to produce hydroxamate-like siderophores, but not catechol-like siderophores, suggesting that these strains are able to bind specific metals [77].

### 2.3. Inhibition of Plant Pathogens

In recent decades, interaction studies have shown that the presence of endophytic microorganisms can increase plant protection against pathogen attacks [81, 82]. Thus, despite of the presence of large numbers of potential phytopathogenic microorganisms inside the plant, most of these interactions remain asymptomatic, due to an elaborate plant defense system [83, 84] and the stability of the microbial community.

Endophytes, including* Methylobacterium* spp., can protect host plants by the synthesis of a large spectrum of antimicrobial molecules [85], nutrient competition with pathogens [86, 87] or by inducing systemic resistance (ISR, Induced Systemic Resistance) [88]. ISR can be induced by volatile organic compounds released from some bacteria [89] and by genes of bacteria that encode plant cell wall degradation enzymes such as glycosidases, cellulases (or endoglucanase) and hemicellulases [90] and pectinase [22, 25] (Figure 2). This mechanism (ISR) has also been reported to induce plant growth and to protect plants against pathogens [22, 81, 91]. Ardanov et al. [92] observed that even at a low density, a* Methylobacterium* sp. IMBG290 inoculum was able to induce potato resistance against* Pectobacterium atrosepticum* by activating the plant antioxidant system; however, at a high density this positive effect was not observed, resulting in susceptibility to the pathogen. In a more recent study, Ardanov et al. [81] evaluated the ability of* Methylobacterium* sp. IMBG290 to induce resistance in several potato (*Solanum tuberosum* L.) cultivars against* P. atrosepticum*,* Phytophthora infestans*, and* Pseudomonas syringae* pv. tomato DC3000, as well as* M. extorquens* DSM13060 in pine (*Pinus sylvestris* L.) against* Gremmeniella abietina.* In addition, the authors observed that plants inoculated with* Methylobacterium* spp. and challenged with the pathogen presented a different endophytic community compared with uninoculated control plants, suggesting that endophyte inoculation may have an effect not only on pathogen establishment but also on plant microbial communities.

Yim et al. [91] reported the induction of defense responses in tomato challenged with* Ralstonia solanacearum* after treatment with four different* Methylobacterium* strains. They verified a reduction in ACC accumulation and consequent reductions in ethylene levels and disease symptoms. In addition, the authors observed an increase in defense enzyme contents, suggesting the potential use of methylotrophic bacteria as biocontrol agents in tomato crops. Furthermore, seed treatment with* Methylobacterium* sp. induced significant protection against* Aspergillus niger* and* Sclerotium rolfsii* pathogens in groundnut under pot-culture conditions [22]. Furthermore, the biocontrol potential of* Methylobacterium* spp. for fungal pathogens such as* Fusarium udum*,* F. oxysporum*,* Pythium aphanidermatum*, and* Sclerotium rolfsii* was also reported under* in vitro* conditions [28]. Kwak et al. [40] described the presence of several genes in* M. oryzae* CBMB20 involved in antimicrobial compound production, such as bacteriocin and 4-hydroxybenzoate as well as 536 genes related to transport, such as amino acid and saccharide transporters, porins, the major facilitator superfamily of permeases, the RND family of efflux transporter MFP subunit proteins, and urea ABC transporters.

In citrus, it was observed that the* Methylobacterium* genus is dominant within branches. Considering the isolation frequency variation, it has been suggested that* Methylobacterium* spp. interact with* Xylella fastidiosa*, the causal agent of CVC (citrus variegated chlorosis) [17, 21]. The presence of the endophytic bacterium* M. mesophilicum* in internal tissues of asymptomatic citrus plants (hosting* X. fastidiosa*) could stimulate the production of compounds that promote the resistance of these plants to* X. fastidiosa* or reduce the growth of this vascular phytopathogen [17], limiting the establishment of* X. fastidiosa* in asymptomatic plants.* In vitro* interaction studies revealed that* M. mesophilicum* SR1.6/6 inhibits the growth of* X. fastidiosa*, while* M. extorquens* AR1.6/2 has no effect on this pathogen [17]. This result was confirmed by Lacava et al. [49], who observed a lower population of* M. mesophilicum* SR1.6/6 and* X. fastidiosa* 9a5c in* Catharanthus roseus* when these bacteria were coinoculated, suggesting that these endophytic and pathogenic bacteria could compete for nutrient and space inside the host plants.

This competition may occur both inside the xylem vessels and inside the insect vectors.* Methylobacterium* spp. plant colonization begins with biofilm formation on roots [13]; the bacteria then colonize the internal tissues of the host plant, including xylem vessels [93]. From the xylem vessels of the host, this endophytic bacterium may be transmitted from plant to plant by sharpshooter vectors, such as* Bucephalogonia xanthophis* [94]. This insect vector is also able to transfer* X. fastidiosa* from infected plants to healthy plants [95], suggesting that these bacteria may interact in different ways inside hosts. This opens a field to search for new strategies for the symbiotic control of pathogens, paratransgenesis. Paratransgenesis means the genetic alteration of symbiotic microbes that are carried by insects to compete with pathogens and control their colonization [94]. Because* M. mesophilicum* was identified between bacterial populations naturally associated with the main sharpshooters responsible for the transmission of* X. fastidiosa* [96], species of the* Methylobacterium* genus have been a promising target for such engineering [93].

In addition, studies that aim to investigate the genes involved in plant-bacteria interactions may expand the understanding of* Methylobacterium*-plant-pathogen interactions and help assess whether there is bacterial genotype specificity to host plants [6, 97]. However, further studies are needed to better understand the molecular and biochemical mechanisms involved in these interaction processes. Ultimately, the discovery of biocontrol agents is the main goal in the search for the reduction of pesticides in agriculture, which may have a negative impact on human health and the environment.

## 3. Bioremediation Using* Methylobacterium* spp. 

*Methylobacterium* spp. are able to biodegrade a variety of organic toxic compounds according to several reported studies. Van Aken et al. [98] observed that* Methylobacterium* sp. strain BJ001 in pure culture was able to degrade several toxic explosives, such as 2,4,6-trinitrotoluene (TNT), hexahydro-1,3,5-trinitro-1,3,5-triazene (RDX) and octahydro-1,3,5,7-tetranitro-1,3,5-tetrazocine (HMX), in 10 to 55 days.* M. extorquens* DM4 also has the ability to degrade a volatile and toxic halogenated solvent, dichloromethane (DCM, CH_2_Cl_2_), which is mainly used and produced by industry [26]. This process occurs by converting DCM into formaldehyde, an intermediate of methylotrophic metabolic growth [99] (Figure 2). The industrial degradation of formaldehyde was reported and tests were performed in a bioreactor, showing that* Methylobacterium* sp. XJLW presents tolerance to 60 g·L^−1^ of formaldehyde and 1,687.5 mg·L^−1^·h^−1^ of degradation rate, being able to degrade 5 g·L^−1^ of formaldehyde [100]. Phenol degradation was also observed. Industrial wastewater can contain high concentrations of phenol; Khongkhaem et al. [101] reported that* Methylobacterium* sp. NP3 and* Acinetobacter* sp. PK1 encapsulated in silica-treated phenol (7500 to 10000 mg·L^−1^) contaminated water efficiently for up to 55 days.

In industrial areas, there are several soil contaminants. In a former industrial site in Italy, the main problem was polycyclic aromatic hydrocarbons (PAHs), a toxic compound resulting from industrial treatment and waste combustion that was present in the studied area. Ventorino et al. [102] showed that* M. populi* VP2, which has several characteristics that promote plant growth, was the isolate best able to degrade PAHs. Another common soil and groundwater pollutant is MTBE (methyl-tert-butyl ether), which is widely used as fuel. It was reported that a reactor able to treat tap water presenting methylotrophic bacteria, including* Methylobacterium* spp., showed a high (over 99.9%) efficiency of MtBE degradation [103]. TCE (trichloroethylene), which is a solvent to remove grease from metal parts, is also a soil pollutant. A combination of a few bacteria, including* Methylobacterium* spp., was able to degrade MTBE and TCE in the presence of heavy metals at a high efficiency (49–182% higher than noninoculated) [104]. These capabilities suggest that bacteria from this genus may be used for the bioremediation of contaminated environments, such as soil and water.

The increasing use of chemical fertilizers and pesticides, results in an increase in heavy metal concentrations in soil, leading to a great environmental impact [105]. Unlike organic contaminants, metals are not degradable and remain in the environment for long periods of time; when present at high concentrations in soil, metals can negatively affect plant metabolism, reducing plant growth [106, 107]. Therefore, a tolerant microorganism can help to bioremediate contaminated water by flocculation [108] and soils by heavy metal immobilization [109], leading to an increase in plant tolerance or even increasing phytoextraction [110] during bacteria-plant interactions. The potential of the methylotrophic genera is shown by their tolerance to high doses of several heavy metals, such as nickel (Ni), cadmium (Cd), cobalt (Co), zinc (Zn), chrome (Cr) [111], arsenic (As), lead (Pb) [27], and mercury (Hg) [104]. Moreover,* M. oryzae* was able to increase Cd and Ni tolerance in tomato plants by decreasing Cd and Ni uptake and promoting plant growth [107].

Haferburg and Kothe [112] reported four main tolerance mechanisms to heavy metals in bacteria: (i) biosorption—the metal binds to the bacterial cell wall, becoming unavailable; (ii) intracellular sequestration—the metal is chelated to a compound inside of the cell; (iii) efflux transporter—the metal is expelled from the cell by a membrane pump [113]; and (iv) extracellular chelation—a chelating compound is pumped out of a bacterial cell, complexing the metal and making it unavailable to other living organisms. An example is the siderophore molecule [114, 115] (previously mentioned in “Section 2.2”), which is able to chelate iron, making it unavailable to pathogens but available to plants.* Streptomyces tenda* F4 was reported to produce siderophores that are able to bind to Cd, decreasing Cd availability to other organisms and decreasing soil availability [114].

Genes related to heavy metal tolerance (uptake and efflux) have been reported in* M. oryzae*, including genes related to the cation efflux system protein CzcA, which are involved in cobalt-zinc-cadmium resistance; several ABC transporters involved in zinc and nickel uptake; copper-translocating P-type ATPase involved in copper resistance; and genes that encode arsenic/arsenate resistance and chromate transport protein. Other transport and secretion systems reported in the* M. oryzae* genome may be related to metal tolerance, for example, the RND family proteins responsible for efflux transport [40].

Therefore, in addition to increasing plant growth and inhibiting plant pathogens, this genus has been reported to increase plant tolerance to heavy metals and to degrade toxic organic compounds in soil, decreasing in this way plant stresses and benefiting even more bacteria-plant interactions. In addition, this genus is able to produce biodegradable plastic and other industrial products, showing its great plasticity and its agricultural and industrial importance.

## 4. Biotechnological Uses of* Methylobacterium* spp. 

Methylotrophic bacteria, in addition to playing a key role in bioremediating contaminated environment and in plant growth, can produce several industrial products and biodegradable compounds. Petroleum-based plastic has a high half-life, causing several problems to the environment. Some bacteria, including* Methylobacterium* spp., are able to produce highly resistant biodegradable plastic that is similar to conventional plastic. Examples of such biopolymers are the biodegradable polyesters polyhydroxybutyric acid (PHB) and polyhydroxyalkanoate (PHA).* M. extorquens* was genetically modified to increase PHB and PHA production using methanol as a substrate [116].* M. organophilum* was also reported to accumulate PHB and PHA [117] under nitrogen limitation while biodegrading methane (a greenhouse gas). Other studies reported that the production of PHB in* Methylobacterium* sp. GW2 could reach 40% w/w of bacterial dry biomass; when supplemented with valeric acid, they also produced the copolyester poly-3-hydroxybutyrate-poly-3-hydroxyvalerate (PHB/HV) [117]. In addition,* Methylobacterium* sp. ZP24 enhanced PHB production when using processed cheese (supplemented with ammonium sulfate) instead of lactose or sucrose in a feed batch [118], and* M. extorquens* AM1 was recently reported to accumulate polyhydroxyalkanoate (PHA) copolymers under cobalt-deficient conditions [119].

Genetic approaches involving PHB biosynthesis were performed, showing that* pha*A,* pha*B, and* pha*C encoding beta-ketothiolase, NADPH-linked acetoacetyl coenzyme A (acetyl-CoA) reductase, and PHB synthase, respectively, were present in the* M. extorquens* AM1 genome and are responsible for PHB synthesis. Furthermore, the authors reported that a* pha*B mutant was not able to grow in methanol, showing that PHB synthesis genes also affect growth in C_1_ and C_2_ compounds in the methylotrophic* M. extorquens* AM1 [120]. In a second study, three more genes,* gap*11,* gap*20 (which encode phasins, granule-associated proteins), and* pha*R (which controls acetyl-CoA flux), were identified to be involved in PHB biosynthesis [121].

Another compound that* Methylobacterium* spp. are able to produce is glyoxylate, an important compound in perfume manufacture and an intermediate in drug and pesticide production [122]. Using genetic engineering, Shen and Wu [122] made a strain able to overexpress the hydroxypyruvate reductase enzyme, a key component in the serine cycle, leading to glyoxylate accumulation.

In agriculture,* Methylobacterium* spp. can contribute to several biotechnological applications. Of all the beneficial characteristics during plant interactions reported above (in “Section 2.2”), Polacco and Holland in 1991 patented (Patent number US5268171) a process that used methanol to select* M. mesophilicum* in the plant and to alter plant metabolism, promoting plant growth. In 1995, Holland and Polacco deposited another patent (Patent number US5512069), in which they coated or impregnated seeds with at least one PPFM to improve seed germination, affirming that PPFM can be used to produce cytokinin. Verginer et al. [123] reported that* M. extorquens* DSM 21961* in vitro* can increase the production of two furanoid compounds, 2,5-dimethyl-4-hydroxy-2H-furanone (DMHF) and 2,5-dimethyl-4-methoxy-2H-furanone, which are responsible for strawberry flavor, showing that the bacterium can influence fruit quality. Nasopoulou et al. [124] reinforced that hypothesis, showing that the expression of the alcohol dehydrogenase enzyme by endophytic bacteria and the flavor components (DMHF) were in the same tissues.

Genetic engineering studies were also performed with* Methylobacterium* spp. A* cry*Aa gene that encodes a protein with activity against lepidoptera from* Bacillus thuringiensis* was cloned and expressed in* M. extorquens*, using the* mxa*F (MDH) promoter, expressing the recombinant Cry1Aa protein and obtaining crystals similar to those formed by the original organism,* B. thuringiensis*, suggesting that this organism could be used to promote plant growth (naturally) and inhibit crop pests (due the transgenic gene) [125]. In a similar way, the *β*-1,4-endoglucanase A gene (encoding EglA) from* Bacillus pumilus* was expressed in* M. extorquens* AR1.6/2 [93], allowing this strain to present cellulolytic activity (ranging from 0.73 to 1.16 U·mL^−1^) [126] and to colonize the plant xylem without inducing disease symptoms in the host plant. This* M. extorquens* AR1.6/2 strain was isolated from the inner tissues of citrus plants infected with* Xylella fastidiosa,* suggesting that this genetically modified* Methylobacterium* species could be used as an agent of symbiotic control [93] because this endoglucanase could degrade a gum produced by* X. fastidiosa*.

## 5. Omics Studies of the* Methylobacterium* Genus

The advancement of molecular biology and the increasing use of next-generation sequencing have enabled the sequencing of many bacterial genomes. Until 2012, only 11* Methylobacterium* genomes were available. Currently, there are 22 sequenced genomes of the* Methylobacterium* genus deposited in the National Center for Biotechnology Information (NCBI) database:* M. extorquens* (AM1, DM4, PA1, DSM13060, CM4);* M. nodulans* ORS2060;* M. populi* BJ001;* M. radiotolerans* JCM2831;* M. mesophilicum* SR1.6/6; and 13 uncharacterized strains (*Methylobacterium* spp.: 4-46, GXF4, MB200, 77, WSM2598, 285MFTsu5.1, 10, B1, B34, 88A, L2-4, EUR3 AL-11 and UNCCL110). The GXF4 strain was the first genome announcement of a plant xylem-associated strain of the* Methylobacterium* genus [43], and SR1.6/6 was the first genome announcement of a* Methylobacterium* strain associated with a tropical plant [127] (Table 2).

Overall, genomes deposited in NCBI have been isolated from different sources, such as air, biogas reactors, plants, and contaminated soils and lakes, showing that the great phenotypic plasticity of those genomes allows the colonization of different niches.* Methylobacterium* genomes are characterized by a GC content between 66.7 and 71.5%, a genome size between 4.6–7.8 Mbp and a plasmid number between 2 and 8 (there is little information about plasmids in the literature) (Table 1). Normally, plasmids encode mainly proteins of unknown function or proteins associated with plasmid-related functions, such as genes for antibiotic resistance and genes for virulence. However, in the AM1 strain some different plasmid functions were reported, such as genes related to cation efflux systems, a cluster of copper resistance genes, a truncated* lux*I gene and* Umu*DC systems [128]. There is also an approximately 130 kb region in the AM1 megaplasmid that is syntenic to a region of similar length in the chromosome of strain DM4. In the CBMB20 strain plasmid, genes encoding DNA polymerase V subunits C and D were observed [40].

Several of these sequenced genomes of* Methylobacterium* strains have been isolated from plants or reported to interact with plants: PA1, DSM13060, ORS2060, BJ001, AM1, GXF4, B1, B34, L2-4, and SR1.6/6, from which four strains were isolated endophytically: SR1.6/6, BJ001, GXF4, and L2-4. Each strain presents a specific interaction with the host plant, and successful colonization may vary according to the species and the stage of plant development [6, 34]. This difference in the success of plant colonization can also be associated with its genome because each strain presents different sizes and numbers of replicons, as well as a specific set of genes, possibly with unknown functions for each strain. The colonization and distribution in the host can also be influenced by plant genotype or by interactions with other associated microorganisms [6, 34].

Vuilleumier et al. [128] analyzed the genomes of two different* Methylobacterium* strains (AM1 and DM4), showing that there were variations in the numbers of insertion elements (IS) and in the organization of the genes, especially those associated with methanol (C_1_) metabolism. Based on these results, the authors suggested that IS is the main mechanism for* Methylobacterium* evolution. On the other hand, a recent study compared the* M. extorquens* strain PA1 isolated from* Arabidopsis* plants to the well characterized* M. extorquens* AM1 strain, showing that the CG contents of PA1 and AM1 strain are quite similar, 68.2% and 68.5%, respectively. Moreover, 90 genes known to be involved with methylotrophy presented more than 95% of identity between these two strains at the amino acid level. Thus, the authors suggested that these two strains have similar specific modules during C1 growth; however, a different growth rate was observed when they used different substrates, which may reflect an adaptation to the niche from which it was isolated [129]. There is a core of conserved genes in the genomes of the* Methylobacterium* genus, which was shown by a study that sequenced six different* Methylobacterium* strains and observed that five of the six strains showed conserved genes involved in photosynthesis, including genes encoding the light-harvesting complex and genes involved in the biosynthesis of bacteriochlorophyll and carotenoids [130], where the core genome consists of 2010 genes and the pan genome of 14,674 genes [40].

Sequenced genomes can be used to identify molecular mechanisms related to plant-*Methylobacterium* or microbe-*Methylobacterium* interactions. Kwak et al. [40] compared the genomes of nine* Methylobacterium* strains and reported that these strains could be divided into three groups with distinguishable features: the first group contains genes for nitrogen fixation (*M. nodulans* and* Methylobacterium* sp. 4-46); the second group has many genes related to plant growth (such as ACC deaminase and phytase) and no nitrogen-fixing genes (*M. oryzae* and* M. radiotolerans*); and the third group (*M. extorquens*) lacked these previous genes.

The genetic and biochemical mechanisms involved in plant-bacteria interactions remain poorly explained, even with many sequenced genomes in databases; one of the most studied bacteria of this genus, the AM1 strain, still needs to be explored, as reported by Ochsner et al. [131] in a review that gathers studies of AM1 strain. Kumar et al. [132] used a proteomics technique to propose a new annotation of a locus function in the AM1 strain. They predicted that the locus MexAM1_META1p1840, previously annotated as hypothetical, had a cytochrome_C3 heme-binding domain and zinc finger domain of HSP40 (Table 2).

The differences between methanol and succinate metabolisms were studied using different approaches. Using microarray techniques, Okubo et al. [133] suggested a connection between methylotrophy metabolism and iron and sulfur homeostasis. Using a proteomic approach, Bosch et al. [134] and Laukel et al. [135] observed the differences in* M. extorquens* AM1 under methylotrophic growth conditions compared to growth on succinate and observed that some proteins were induced depending on growth conditions. The metabolite profiles under the previous conditions were also analyzed by Guo and Lidstrom [136], who reported several different metabolites in cells grown on methanol and on succinate, although only 13 matched to the mass spectra database. Metabolomics studies of* M. extorquens* AM1 were also performed in other conditions, comparing C2 and C4 metabolism, using ethylamine (C2) and succinate (C4) as carbon sources. Both liquid chromatography-tandem quadrupole mass spectrometry (LC-MS/MS) and two-dimensional gas chromatography-time-of-flight mass spectrometry (GCxGC-TOF-MS) were used and were able to validate the metabolites' qualifications. The results showed that the abundance of 20 intermediates varied under different metabolisms, evidence that there are differences not only between the C1 and C4 but also between the C2 and C4 pathways [137].

Schneider et al. [138] reported that the* M. extorquens* AM1 ethylmalonyl-CoA pathway functionally replaces the glyoxylate cycle (isocitrate lyase) during growth on acetate, suggesting that such an organism can adapt its metabolism to changes in carbon source availability. A proteomic study of* M. extorquens* during the colonization of the phyllosphere of* Arabidopsis thaliana* described the upregulation of proteins from the antioxidant system and underscored the increased expression of the PhyR regulator, which is important for the colonization of the phyllosphere [59]. Later, Francez-Charlot et al. [139] elucidated PhyR regulation using a transcriptomic analysis, showing that PhyR regulates gene expression though protein-protein interactions and that NepR negatively regulates PhyR by the sequestration of the ECF sigma factor. Evidence of differences from other well-known general stress regulators, such as *σ* ^*S*^ and *σ* ^*B*^, suggests that Alphaproteobacteria has a novel mechanism of general stress response.

In addition to their interactions with plants,* Methylobacterium* strains are able to degrade several organic compounds, including dichloromethane and chloromethane [26, 127, 140]. Different approaches comparing dichloromethane-degrading strains to the* M. extorquens* AM1 reference strain have reported that rearrangements and horizontal gene transfer resulted in great genomic plasticity [128]. In addition, authors observed that the success of horizontal transfer of the* dcm*A gene (which confers the ability to grow on dichloromethane) was not related to the phylogeny of the parental strain, but to the adaptation to the stress and the metabolic disruption resulting from the acquisition of a new enzyme or pathway [141].

In this way,* Methylobacterium* strains can be used to increase plant growth (producing auxins and siderophores, fixing nitrogen, decreasing ethylene production, and solubilizing phosphorus), to inhibit plant pathogens and to induce systemic resistance in plants. In addition, these strains can act in bioremediation processes that degrade toxic organic compounds, increasing plant tolerance and possibly increasing the phytoremediation of inorganic compounds. All of these characteristics show the bacterial potential in agriculture.

Several beneficial plant growth-promoting processes have already been reported in* Methylobacterium* strains, as mentioned above; however, more studies are needed to make it possible to elucidate all of those interaction processes at the biochemical and molecular levels. Genomic, transcriptomic and proteomic approaches enable us to study the structure and infer the functions of different metabolic pathways as well as to understand some integrated aspects of microorganism biology, that is, to correlate gene sequences, expression patterns, protein functions, and interactions. Thus, these approaches will provide us with essential clues to understand the evolutionary history of biological systems and to support biotechnological applications in different areas of interest.

## 6. Concluding Remarks

There has been a lot of research in* Methylobacterium* genus since its first reported in 1976 [1], which was largely increased after the use of next generation sequencing technology, enabling us to study the genes present in the genome, the expressed genes, and the proteins and metabolites produced in different conditions allowing us to understand some mechanisms involved during plant interaction, explaining how these PPFM bacteria are able to induce plant growth, decreasing the establishment of plant pathogens and plant stress, as well as comprehend the role of these bacteria during bioremediation of contaminated soils. Unfortunately, in the present review we were not able to include all published articles of this subject; for more information, there are other* Methylobacterium* reviews [55, 131]. Moreover, there are innumerous microorganism interactions occurring in the plant environment that still need to be elucidated, requiring more research.

## Figures and Tables

**Table 1:** Sequenced genomes (deposited in the NCBI database) of *Methylobacterium* strains and their genomic features.

Organism	Strain	GenBank assembly ID	Isolation source	Key characteristic(s)	Genome size (Mpb)	CG content (%)	Gene	Number of plasmid	Sequencing institute
*M. extorquens *	AM1	GCA_000022685.1	Air	Growth on methylamine or methanol	6.88	68.7	5065	4	University of Washington Genome Center

*M. extorquens *	DM4	GCA_000083545.1	Soil contaminated with halogenated hydrocarbons	Chloromethane degrader	6.12	68.1	5851	2	Genoscope

*M. extorquens *	PA1	GCA_000018845.1	*Arabidopsis thaliana *	*Arabidopsis thaliana* epiphyte	5.47	68.2	4956	⋯	DOE Joint Genome Institute

*M. extorquens *	CM4	GCA_000021845.1	—	—	6.18	68.2	5463	2	DOE Joint Genome Institute

*M. extorquens *	DSM13060	GCA_000243435.2	Meristems of *Pinus sylvestris *	*Pinus sylvestris* epiphyte	6.67	68.30	6894	⋯	DOE Joint Genome Institute

*M. mesophilicum *	SR1.6/6	GCA_000364445.1	*Citrus sinensis* branches	Citrus endophyte	6.2	69.47	6052	⋯	Rede Paraense de genômica e proteômica

*M. nodulans *	ORS2060	GCA_000022085.1	from root nodules from the legume *Crotalaria *	Nonpigmented, fix nitrogen, *Crotalaria* nodulating	7.78	68.9	7765	7	DOE Joint Genome Institute

*M. populi *	BJ001	GCA_000019945.1	*Populus* sp.	*Populus deltoides* x nigra DN34 endophyte	5.80	69	5492	2	DOE Joint Genome Institute

*M. radiotolerans *	JCM2831	GCA_000019725.1	—	Radioresistant strain, fix nitrogen, nodulate plants	6.08	71.5	5839	8	DOE Joint Genome Institute

*Methylobacterium* sp.	4-46	GCA_000019365.1	—	*Lotononis bainesi* nodulating, photosynthetic	7.66	71.4	7145	2	DOE Joint Genome Institute

*Methylobacterium* sp.	GXF4	GCA_000272495.1	grapevine xylem fluids	Endophytic from xylem fluids	6.12	69.6	5976	⋯	Rochester Institute of Technology

*Methylobacterium* sp.	MB200	GCA_000333655.1	biogas reactor	—	5.77	68.9	5038	⋯	College of Life Science and Technology, Guangxi University

*Methylobacterium* sp.	77	GCA_000372825.1	Lake Washington	—	4.66	66.7	4108	⋯	DOE Joint Genome Institute

*Methylobacterium* sp.	WSM2598	GCA_000379105.1	*Lotononis bainesii *	Root nodulating bacteria, bacteria-plant-soil association	7.67	71.2	6631	⋯	DOE Joint Genome Institute

*Methylobacterium* sp.	285MFTsu5.1	GCA_000383455.1	—	Bacteria-plant association	6.62	71	5970	⋯	DOE Joint Genome Institute

*Methylobacterium* sp.	10	GCA_000519085.1	Lake Washington	—	4.98	66.7	4285	⋯	DOE Joint Genome Institute

*Methylobacterium* sp.	B1	GCA_000333255.1	rice shoot	—	5.91	69.6	—	⋯	Kazusa DNA Research Institute

*Methylobacterium* sp.	B34	GCA_000333475.1	rice shoot	—	6.93	70.4	—	⋯	Kazusa DNA Research Institute

*Methylobacterium* sp.	88A	GCA_000376345.1	Lake Washington	—	4.89	67.1	4274	⋯	DOE Joint Genome Institute

*Methylobacterium* sp.	L2-4	GCA_000454305.1	*Jatropha curcas* L.	*Jatropha curcas* L. endophyte	6.8	70.8	6255	⋯	Temasek Lifesciences Laboratory

*Methylobacterium* sp.	EUR3 AL-11	GCA_000526475.1	—	thermal adaption and carbon metabolism in permafrost	7.21	71.1	6670	⋯	DOE Joint Genome Institute

*Methylobacterium* sp.	UNCCL110	GCA_000745415.1	—	bacteria-plant association	6.61	69.7	—	⋯	DOE Joint Genome Institute

**Table 2:** Omics studies in the *Methylobacterium* genus.

Organism	Approach	Findings	References
*M. extorquens AM1 *	Proteome	Identify a PhyR stress regulator during phyllosphere colonization using 2D analysis.	[59]
*M. extorquens AM1 *	Proteome	Proteomic comparison under single carbon (methanol) and multicarbon (succinate) growth in a gel free quantitative proteomic assay.	[134]
*M. extorquens AM1 *	Proteome	Cytosolic protein differentially modulated under single carbon (methanol) and multicarbon (succinate) growth.	[135]
*M. extorquens AM1 *	Proteome	Compare wild type with *M. extorquens* AM1 lacking isocitrate lyase (the key enzyme in the glyoxylate cycle) during growth on acetate, which was replaced by ethylmalonyl-CoA pathway.	[138]
*M. extorquens DM4 *	Proteome	Differential proteomic analysis of cultures grown with DCM and with methanol elucidates growth metabolism in the presence of DCM.	[26]
*M. extorquens CM4 *	Proteome and genome	Comparison of growth with one-carbon substrates: chloromethane and methanol, reporting the genes required for chloromethane utilization.	[140]
*M. extorquens AM1 *	Proteogenome	Refined the annotation of protein coding genes and discover genes in *M. extorquens* AM1 genome.	[132]
*M. extorquens AM1 *	Metabolomic	Analyze the metabolites produced during single carbon (methanol) and multicarbon (succinate) growth, providing clues to new pathways that are specifically linked to C1 metabolism.	[136]
*M. extorquens AM1 *	Metabolomic	Metabolites produced by *M. extorquens* AM1 grown on two carbon sources, ethylamine (C2) and succinate (C4) using LC-based and GC-based methods showing differences in in pathways linked to C2 and C4 metabolism.	[137]
*M. extorquens AM1 *	Transcriptome	Validate a microarray plataform comparing genes expressed during single carbon (methanol) and multicarbon (succinate) growth, pointing candidate genes in C1 metabolism.	[133]
*M. extorquens AM1 *	Transcriptome	Elucidates the regulation of general stress regulator (PhyR) using microarray.	[139]
*M. extorquens AM1 e DM4 *	Genome comparison	Genome comparison of *M. extorquens* strain AM1 (reference strain) and the dichloromethane-degrading strain DM4 concluding that rearrangements and horizontal gene transfer resulted in a great genomic plasticity.	[128]
*Methylobacterium *strains	Genome announcement	Six *Methylobacterium* strains adapted to different plant-associated niches and environmental: *M. extorquens* (PA1, CM4. BJ001), *M. radiotolerans* (JCM2831), *M. nodulans* (ORS2060), and *Methylobacterium* sp. 4-46	[130]
*M. mesophilicum SR1.6/6 *	Genome announcement	Endophytic bacterium isolated from a surface-sterilized *Citrus sinensis*.	[127]
*Methylobacterium* sp. *GXF4 *	Genome announcement	A xylem-associated bacterium isolated from *Vitis vinifera* L. grapevine.	[43]
